# Combination of Ceramic Laser Micromachining and Printed Technology as a Way for Rapid Prototyping Semiconductor Gas Sensors

**DOI:** 10.3390/mi12121440

**Published:** 2021-11-25

**Authors:** Nikolay Samotaev, Konstantin Oblov, Pavel Dzhumaev, Marco Fritsch, Sindy Mosch, Mykola Vinnichenko, Nikolai Trofimenko, Christoph Baumgärtner, Franz-Martin Fuchs, Lena Wissmeier

**Affiliations:** 1 Micro and Nanoelectronics Department, MEPhI (Moscow Engineering Physics Institute), National Research Nuclear University, 115409 Moscow, Russia; kyoblov@mephi.ru (K.O.); psdzhumaev@mephi.ru (P.D.); 2 Fraunhofer IKTS Institute, 01277 Dresden, Germany; marco.fritsch@ikts.fraunhofer.de (M.F.); Sindy.Mosch@ikts.fraunhofer.de (S.M.); mykola.vinnichenko@ikts.fraunhofer.de (M.V.); Nikolai.Trofimenko@ikts.fraunhofer.de (N.T.); christoph.baumgaertner@ikts.fraunhofer.de (C.B.); 3 KERAFOL Keramische Folien GmbH & Co. KG, 92676 Eschenbach, Germany; franz-martin-fuchs@kerafol.com (F.-M.F.); Lena-Wissmeier@kerafol.com (L.W.)

**Keywords:** laser micromachining, printing technology, platinum ink, thin ceramic membrane

## Abstract

The work describes a fast and flexible micro/nano fabrication and manufacturing method for ceramic Micro-electromechanical systems (MEMS)sensors. Rapid prototyping techniques are demonstrated for metal oxide sensor fabrication in the form of a complete MEMS device, which could be used as a compact miniaturized surface mount devices package. Ceramic MEMS were fabricated by the laser micromilling of already pre-sintered monolithic materials. It has been demonstrated that it is possible to deposit metallization and sensor films by thick-film and thin-film methods on the manufactured ceramic product. The results of functional tests of such manufactured sensors are presented, demonstrating their full suitability for gas sensing application and indicating that the obtained parameters are at a level comparable to those of industrial produced sensors. Results of design and optimization principles of applied methods for micro- and nanosystems are discussed with regard to future, wider application in semiconductor gas sensors prototyping.

## 1. Introduction

A metal oxide (MOX) sensor is a good example for rapid prototyping of functionally complete MEMS devices in compact packages for surface mount devices (SMD package). The MOX sensor is not only a complex MEMS product, but it has also a complex and specific package. The general construction of the gas-sensitive metal oxide sensor [1] is a combination of the following parts responsible for specific functions:

Microhotplate, which is responsible for the level of power consumption and parameters of temperature cycling regime of the metal oxide gas sensor;

Chemical composition of the layer of nanostructured metal oxide sensitive to the gas, which is responsible for the sensitivity to specific gases;

Package, responsible for the functional applications in which the sensor can be used (for example, by the level of explosion protection or vibration resistance).

Despite the existing market of companies with a large production of MOX sensors [2,3,4,5] for gas analytics, there are not all-encompassing solutions for sensing tasks (breath tests [6], security application [7], and the food industry [8]). That is why fast prototyping methods are becoming relevant, since they allow the production of small sensor lots with a customized design and layout. The use of flexible prototyping methods for the production of metal oxide sensors offers deepened system integration at the instrument level as well as reduced production time and cost for specific semi-customer gas analysis device. This approach is challenged by already existing MEMS solutions, mainly based on silicon clean room technology, which is demonstrated for example in article [9] for a prototype of a gas pre-concentrator. The scaled experimental integration of MOX sensor arrays needs the development of an individual plastic package [10]. Larger serial scale products like gas sensor modules for indoor air quality monitoring, based on MOX sensors in a micro-assembly based on several silicon chips, use a microcontroller for a normalized digital signal [11,12]. These examples illustrate that gas sensing applications can vary widely by environmental application conditions, but there is nearly no flexibility to realize adapted prototype sensors in smaller, cost-effective numbers.

Regarding smart sensors and electronics for agro-industrial systems, commercially available sensor solutions show drawbacks concerning continuously and cost-efficient measurements as well as long-term stability in corrosive gas environments. For these cases, we developed MOX gas sensors for industrial use under harsh environmental conditions [13], where we combine ceramic substrates and packaging materials with ceramic MEMS technology [14]. 

The approach to manufacture MOX sensors with 3D prototyping technologies presupposes the availability of materials and methods. In our work, we demonstrate that this approach to the fast prototyping of MOX sensors is possible and the sensors’ properties are comparable in terms of the gas sensing ability to commercial sensors. Monolithic ceramics based on zirconium oxide [15] and aluminum oxide [16] were chosen as the materials for the MEMS and SMD package, metallizations were aerosol-jet printed by platinum [17] and silver [18] inks, and a gas-sensitive MOX layer was the synthesis on the basis of tin dioxide [19]. The construction of the sensor was designed based on the concept of finding a balance between the resolution of the used equipment and acceptable thermal characteristics [20].

## 2. Materials and Methods

**Figure 1:**
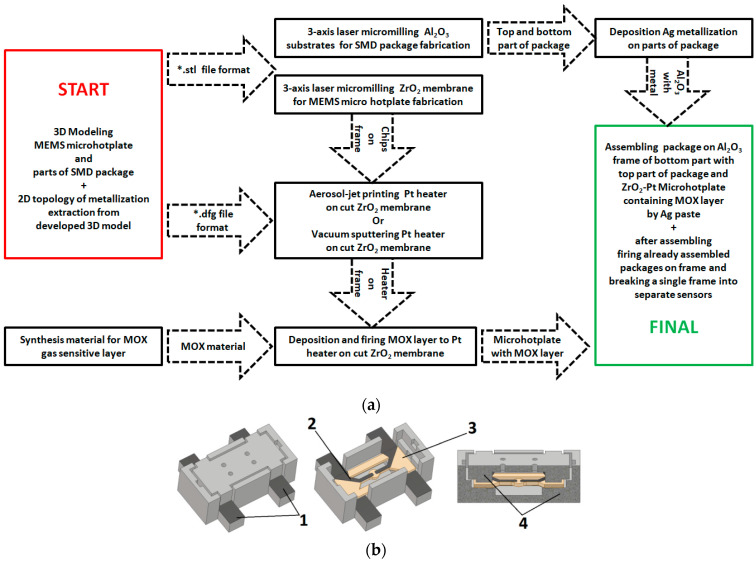
Digital technological flow used for rapid prototyping metal oxide (MOX)gas sensors: (**a**) Full flowchart for rapid prototyping of MOX gas sensors; (**b**) 3D model of gas sensor in the SOT-23 package using for fabrication: 1—Ag metallization; 2—Pt metallization; 3—YSZ membrane; and 4—Al_2_O_3_ ceramics.

To fabricate all parts of the gas sensor, we used a digital technological flow (as presented in Figure 1a). We developed a 3Dmodel of the sensing device by using 3D modeling software. As a result, the file was a STL format (as presented in Figure 1b). To reduce the sensor cost, Al_2_O_3_ monolithic ceramic was used to manufacture the package of the sensor. The SOT-23 package (size is 3.0 × 1.4 × 1.0 mm^3^), which is widely known in electronics, was used as a form factor, since it makes it possible to dissipate a heating power up to 350 mW at room temperature [21]. A special 20 W fiber laser with tunable pulse duration in the range of 50–200 ns and a wavelength of 1.064 μm, controlled by specially produced software [22], was used to fabricate different parts of the developed sensor. This approach allowed us to combine the process of micromilling with a digital comparison of the fabricated devices, its geometrical parameters within the3D model, and the achieved quality after the fabrication process.

### 2.1. Fabrication Ultra Thin Zirconium Oxide Membrane

**Figure 2:**
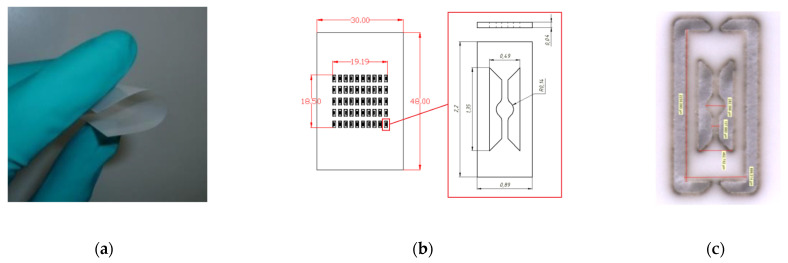

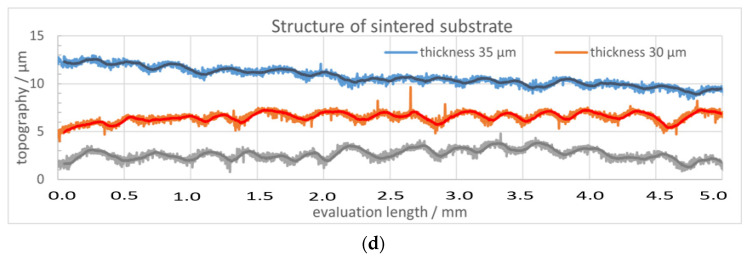
The following displays an ultra-thin zirconium oxide membrane: (**a**) Demonstration of flexibility of 30 µm (5 × 5 cm^2^) thin 3YSZ substrate, (**b**) sketch of 3YSZ membranes for cutting by laser to single microhotplate chips; (**c**)optical view of a 3YSZ microhotplate chip attached to a ceramic frame; and (**d**) the surface roughness measurement of the YSZ membrane after sintering.

Yttria-stabilized zirconia (3YSZ) powder was used for the preparation of casting slurries and tape casting of thin tapes (<50 µm) (see Figure 2a). After thermal treatment, the substrates were characterized by density, thickness, flatness, roughness, and mechanical bending stability. To reduce the thermal mass of the hotplate, the ceramic substrate was cut (see Figure 2b) by a developed micromilling process to realize free-standing membranes of 280 µm in diameter. This step allowed us to reduce the heat capacity of the microhotplate.

Before laser cutting, the YSZ ceramic membrane needs to be characterized by a profilometer to determine membrane roughness (result is presented in Figure 2d). This can significantly save time, since having such profilometer results in advance can be used to determine which kind of metallization type is suitable for deposition on the membrane device.

### 2.2. Fabrication Platinum Printing Microhotplate

**Figure 3:**
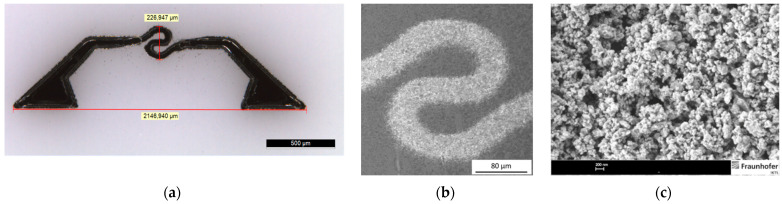
Platinum-printed microhotplate: (**a**) Optical image with dimension (red lines: vertical ~227 µm and horizontal ~2147 µm) platinum microhotplate printed by an aerosol-jet system on a thin 3YSZ substrate before firing; (**b**) SEM image of the aerosol-jet printed heater after sintering; and (**c**) SEM image of milled glass powder <500 nm, which was used for the Pt-glass ink.

Pt-glass-composite inks (30 wt. % solid content) were synthesized and ink properties like viscosity, surface tension, and sedimentation stability were characterized. A synthesized glass powder, based on the oxides of boron, tin, calcium, and silicon, was milled by high-energy ball milling to achieve a particle size <0.5 µm and dispersed in the Pt-ink. Printing tests were carried out by an Optomec M3D printer with a 150 µm nozzle (aerosol-jet). The microheater layout was 2.0 × 0.5 mm^2^ in size with a 40 µm line width in the inner hot spot (see Figure 3a). The printed heaters were sintered in a box furnace at 650 °C. The printed heaters were characterized after sintering by film thickness, resistivity, and microstructure (SEM).

The prepared Pt-glass ink shows a viscosity of 6 cP and surface tension of 24 mN/m, which is compatible with aerosol-jet printing. The quality of printed samples in terms of film homogeneity and printed line width (~40 µm) in the center of the heater layout was quite good (see Figure 3a). To increase the adhesion of the printed Pt film on the smooth 3YSZ surface, glass powder was successfully prepared by high-energy milling with particle size <500 nm (see Figure 3b). Larger particle sizes are an issue for aerosol-jet printing, since the largest particles in the ink should be limited to be below 1 µm in size. Industrial standard glass frits and powders are typically in the range of 4 to 20 µm size, which cannot be used without special milling techniques. The sintered Pt-heater showed a resistance of 30 to 40 Ohm, which is near the desired target of 10 Ohm. However, we observed issues in ink-drying behavior with achieving a homogenously dense Pt-film (thickness 0.5 to 1 µm). This led to local areas with thin spots and crack formation. It can be expected, that with optimized Pt-inks and drying regimes, a qualitative functionable Pt-heater can be achieved. Alternatively, a thin Pt-heater can be produced by vacuum-thin film technology.

### 2.3. Fabrication Platinum Sputtering Microhotplate

**Figure 4:**
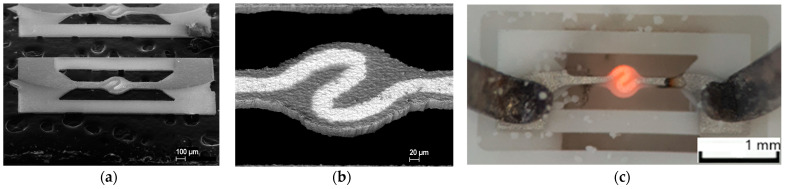
Platinum-sputtered microhotplate: (**a**) SEM image of platinum-sputtered microhotplate on a thin 3YSZ substrate; (**b**) SEM image of hot spot for platinum microheater sputtered by magnetron on a thin 3YSZ substrate; and (**c**) a platinum microheater on 3YSZ membranes under melting point technique evaluation.

As an alternative and reference technology, microhotplates were fabricated by magnetron sputtering of a pure platinum target. The layout of a microhotpalate was 60 µm in width, 0.5 µm in thicknesses, and 10 Ohm in target resistance at 20 °C (see Figure 4b). The layout was formed by using a shadow mask on a 48 × 30 mm^2^ size substrate. Prior to Pt sputtering, segments of microhotplate chips were prepared by laser micromilling. Each substrate possesses 50 miniaturized samples of thin membrane-type microhotplates. The process of double side sputtering took a few hours for deposition of platinum metallization on both substrate sides. The distance between the electrodes to the gas sensitive layer was chosen to be 250 μm. The layout for gas sensitive electrodes was chosen based on the experiences of past works, where we tried to demonstrate gas-sensitive MOX layers based on the SnO_2_ material with a standard resistance of 1 MΩ at 450 °C in pure air [23].

### 2.4. Fabrication Ceramic Package for MOX Sensor

The prototyping SMD packages was realized by laser micromilling technology with Al_2_O_3_ ceramic substrates (96% alumina) in the size of 48 × 60 mm^2^ in two thicknesses of 0.5 and 1 mm. The software and hardware developed for the micromilling process is described in more detail in the publication [24]. At the stage of 3D modeling, it is necessary to add jumpers to the model, which will hold it in the substrate (frame) array. The point of contact of the jumper with the 3D model depends on the size of the model; for our models presented in Figure 1, it is a pyramid with a vertex in the form of a square of 150 × 150 microns (where it is attached to the chip). The preparation time for milling a 3D object of this size with a specialized software takes less than a minute. The process of laser micromilling of Al_2_O_3_ ceramics was carried out at a speed of ~40 mm^3^/h. Depending on the required product quality, the milling speed can be changed up or down. After starting micromilling, the process can be paused at any time to view the milled object using a microscope with 400–2000× magnification or to measure the roughness/height of the milled layer using a point laser profiler integrated into the adaptive laser micromilling unit, and then continue milling from the stop point. Time of fabrication of both the part of the SOT-23 package is 18 min (4 min takes a milling cap part of the package from a 0.5 mm substrate and 14 min takes a milling bottom part of the package from a 1.0 mm substrate). After laser micromilling, Ag ink was deposited on the bottom part of this SMD package to realize package metallization (e.g., soldering contact pads).

**Figure 5:**
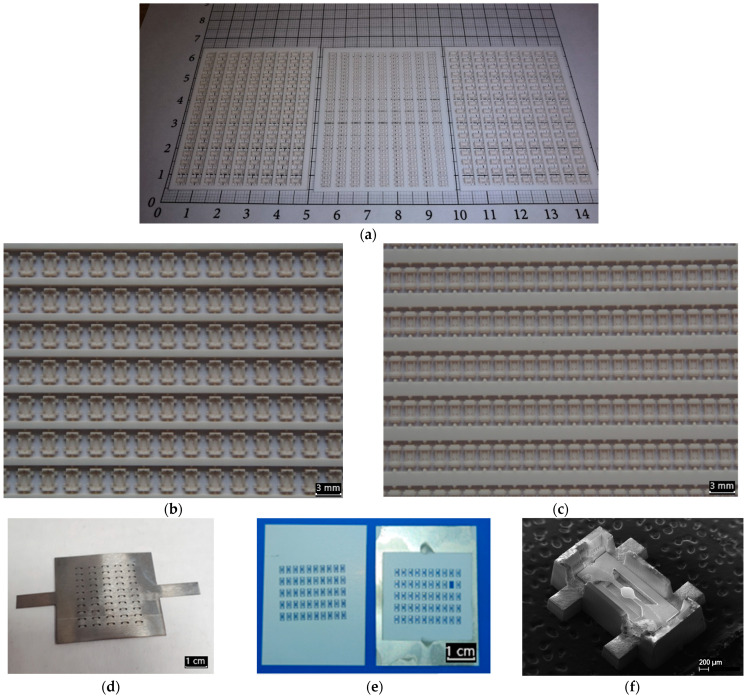
Scaling of laser micromachining fabrication of MOX sensor packages and 3YSZ microhotplates: (**a**) Three ceramic substrates containing a maximum density of SOT-23 packages parts—in the middle, a 0.5 × 60 × 48 mm^3^ substrate containing frame 9 × 35 = 315 pcs. caps of package and on the outside, 1.0 × 60 × 48 mm^3^ substrates containing each frame 9 × 17 = 153 pcs. (total for two substrate 306 pcs.) bottom of the package; (**b**) magnified optical image of the package bottoms on a ceramic frame from the middle substrate on (**a**); (**c**) the magnified optical image of the package bottoms on a ceramic frame from outside substrates on (**a**); (**d**) shadow mask for Pt vacuum sputtering fabricated by laser cutting in the same process as 3YSZ membranes; (**e**) 3YSZ membranes (30 × 48 mm^2^) after laser cutting on 50 pcs. single chips for microhotplates (left) and one after process of shadow mask Pt vacuum sputtering(right); and (**f**) SEM image of MOX sensor before final gluing the cap of package.

Due to the possibility of the parallel processing of a lot of individual small MOX sensor components, which are arranged as an array on a larger ceramic substrate, the developed micromilling technology is a very powerful tool to realize such miniaturized ceramic MOX sensor components economically. Figure 5 illustrates the fabrication row of a 306-sensor package out of only 3 ceramic substrates with a size of 48 × 60 mm^2^. The high packing density of the components on the ceramic substrate is inaccessible with other technologies and is only possible due to the high-quality ceramics developed by Kerafol, which maintain their high mechanical strength during the laser ablation process.

## 3. Results

**Figure 6:**
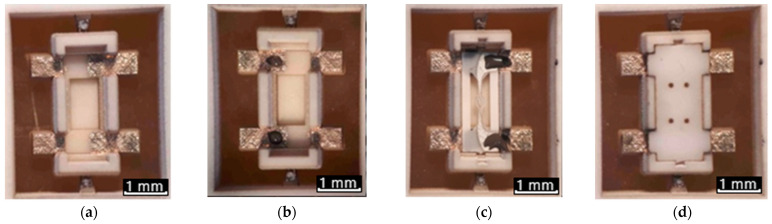
Assembling of parts of a sensor to form the SMD package SOT-23: (**a**) Bottom parts of gas sensor package after laser micromilling and Ag metallization deposition, (**b**) deposition of Ag-Pt paste on bottom parts of gas sensor package for gluing the YSZ membrane with microhotplate; (**c**) deposition of Ag-Pt paste on the YSZ membrane with microhotplate for gluing top parts of the package; and (**d**) already assembled gas sensor after firing.

After fabrication of all MEMS sensor parts and finishing all specific operations for deposition of the MOX gas sensitive layer (we use a drop-coating technique), the sensors were assembled step by step, which is shown in Figure 6. The assembly is carried out in hand-made mode and ends by firing all parts of the sensor (membrane with a microhot-plate, as well as the bottom and top part of the package) in a furnace in parallel, which turns the sensor part into a monolithic ceramic package. The SEM photo of a fully assem-bled and already fired sensor is shown in Figure 5. We would also like to draw attention to the high stability of the used materials—they were all exposedto high mechanical stress, caused by handmade manipulation and high-temperature processing. 

**Figure 7:**
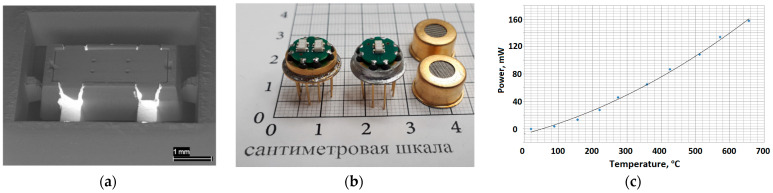
Result of sensor testing: (**a**) SEM image of fabricated sensor after final firing of Ag metallization on contact pads and before detachment from the ceramic substrate frame; (**b**) SOT-23 packages soldered on a PCB mounted in the metal-glass TO-8 package (diameter of top part is ~11 mm [27]), scale given in centimeters; and (**c**) power consumption for the MOX sensor in a SOT-23 package vs. working temperature.

Sputtered Pt-heaters with an appropriate resistance in a range of 10 Ohm were tested by a micro-melting technique [25] at temperatures range up to 600 °C. To conduct these tests, we used a 50 µm grain size polyamide micro powder [26] with a melting point of 180 °C to understand the dependence of power consumption on working temperature at the surface of a microhotplate, which is presented in Figure 4c. The platinum metallization on the YSZ membrane has a temperature coefficient of resistance of ~3490 ppm/°C. The temperature-power consumption relationship of the microhotplate is presented in Figure 7c. The power consumption was calculated by using the applied voltage and current. The power consumption testing shows 95 ± 5 mW for a 450 °C working temperature from sample to sample, fabricated on the same YSZ membrane (see Figure 2). This fluctuation is rather low, considering the small dimension of the sensor package and the higher termoconductivity of the Al_2_O_3_ package compared to the YSZ membrane. The sensor power consumption tests were done in final assembled package (SEM photo present on Figure 7a), which was soldered to a standard Printed Circuit Board (PCB) with coating electroless nickel and immersion gold process (see Figure 7b).

**Figure 8:**
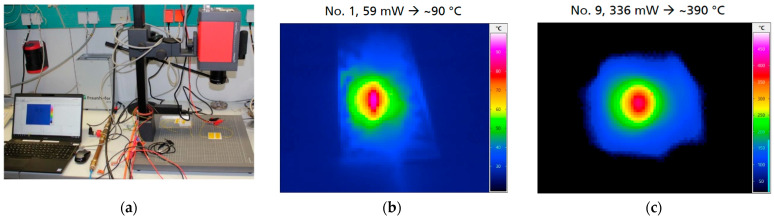
Result of platinum-printed microhotplate testing: (**a**) Photo of testing installation with an IR camera and placement of the Pt-heater chip underneath; (**b**) IR image at 90 °C working temperature for microhotplatehot spot with power consumption of 59 mW; and (**c**) IR image at 390 °C working temperature for microhotplate hot spot with power consumption of 336 mW.

The characterization of power consumption of aerosol-jet printed microhotplates was done by an IR camera (setup present on Figure 8a) and shows a 380 ± 20 mW power consumption for 450 °C (recalculated relativity 336 mW for 390 °C by Figure 8c). This value is within the technical limit for the standard SOT-23 package power dissipation at room temperature. In the case of using printed microhotplates at more than 450 °C working temperature, special conditions for the PCB topology, which are used for SOT-23 package soldering, are needed. Directly under the package are metal traces that act for heat staking (in this way it is possible dispose of up to 1500 mW thermal power). The difference in power consumption can be explained by the thickness of the metallization of the microheater—for a vacuum sputtered heater, it is 0.5 µm and for a printed heater, it is 1.5 µm, which results in a difference of power by a factor of three.

## 4. Discussion

The experience of the described technology demonstrates that this approach that is taken by qualified specialists in the field of 3D modeling and laser milling, is a fantastic speed of design and manufacture of ready-to-use ceramic MEMS devices that can be reached. The production time of a set of three parts, namely, the MEMS microheater, bottom, and cover of the SMD package (3D model is presented in Figure 1, produced package is shown in Figure 4), is fabricated within 3 to 15 min, depending on the complexity of 3D models and the type of used ceramic (the time of laser micromachining is significantly affected by the heat capacity of the ceramic materials). The limiting point of this technology chain is the drying and sintering steps of printed Pt and Ag metallization in a muffle furnace, which requires at least one hour, but could be done for all fabricated samples in parallel. In addition to the production flexibility based on 3D modeling, the main advantage of this technological process is the use of ceramic material. Ceramics used for the laser micromilling allow a broadening of the range of working temperatures of the metal oxide sensor for up to 800 °C and can also increase the annealing temperature of the gas-sensitive metal oxide layer up to 1000 °C (in comparison this temperature for silicon technology, which is only 700 °C [28]).

The results of this work demonstrate a fully functional device using the above rapid prototyping technology for semiconductor gas sensors. The question revolves around the flexible applicability of these results. The flexibility of this approach can be suggested by using various MOX gas-sensitive layers, using different technology methods—thin-film (vacuum) and thick-film (atmosphere pressure). For thin-film MOX gas-sensitive layers, thicknesses starting from 0.1 µm up to 10 µm [29] are typical, which can be obtained by various methods of vacuum deposition—PLD [30], DC [31], or RF [32] magnetron sputtering, ALD [33], LPCVD [34], but in the main parts of experiments using MOX layers thicknesses of several micrometers obtained by magnetron sputtering [35]. For thick-film technology, thicknesses from 5 µm up to 50 µm [36] are typically obtained through the use of several processes—screen printing [37], drop coating [38], spin coating [39], spray pyrolysis deposition [40], and inkjet printing [41].

If we consider thin-film technology, then the absence of serious roughness of the substrate, on which the film is deposited, is considered as a good criterion for the deposition of a MOX gas-sensitive layer. This type of technology mainly uses thin silicon membranes with a hot spot area in a diameter of around 100–500 μm [34]. In our case, the YSZ membrane roughness of less than 0.4 μm (profilometry test is presented in Figure 2d) allows vacuum deposition without fear of the formation of defects like “isolated islands” or “punctures” of a thin film that do not contribute to the film conductivity of the MOX material. Moreover, the use of thin-film platinum contacts, deposited by magnetron, with a thickness of 0.4 µm allows the following deposition of a MOX gas-sensitive layer on top of these contacts, as reported in [29] (combination 0.1 µm MOX layer with 0.4 µm Pt electrode). In addition, varying the distance between the contact electrodes allows the increase or decrease of the nominal value of the resistance of such gas-sensitive layers, which also has a positive effect on the overall functionality of the MOX sensor. For thin-film technology, the temperatures of technological annealing of gas-sensitive layers do not play a significant role, as a rule, they are not higher than the operating temperatures, since the initial target already contains all the necessary compositions of materials. In addition, the use of a shadow mask for the deposition of platinum electrodes or deposition of a gas-sensitive layer cut from a metal foil on the same laser unit (result is presented if Figure 5d) and according to the same 3D model as the sensor is manufactured, allows one to have a very clean and fast process of applying thin films, without the additional operations of washing the photo resist or post-processing by chemical etching. 

If we consider printing technologies, the flexibility in design options, for deposition MOX gas-sensitive layers, become even wider. First, the high stability of the ceramic materials used in the developed microhotplate at high temperatures allows for the use of a wide range of technologies, up to the most extreme technological processes for synthesis MOX gas sensitive materials—for example, flame pyrolysis [42]. Additional flexibility is given by using a hot spot layout of microhotplate by a “circle” type, which allows the use of drop coating or inkjet printing technologies for the deposition of MOX for long-term stability and methods of deposition using screen printing [43]. The hot spot size of approximately 280 µm and the high mechanical strength of the YSZ membrane allows for easy screen printing. Of course, screen printing as a rule, due to the mass production of the product, is more suitable for medium-scale production than for prototyping, but it is still applicable and if one wants to begin the mass production of the developed gas sensor, the step from prototype to serial sample will be very short. As mentioned, the same laser unit can cut the foil of the shadow mask, which is compatible in design to the screen-printed deposition MOX gas-sensitive layer according to the already-developed 3D model for sensor manufacturing. 

However, in addition to the flexibility of the process, the presented technology has a significant drawback inherent in all prototyping systems—this is the time of sample production, which linearly depends on the number of samples produced and is difficult to vary and reduce. To reduce the time for manufacturing large batches of sensors, it is necessary to use a group production method that is outside of prototyping technology and is its antagonist. The only thing that makes it possible to reduce the production time of the prototype is to reduce its size, but there are also limitations in the form of hand-made operations for gluing the membrane and assembling parts of the SMD package parts in a complete device. Our experience also shows that manual operations are greatly accelerated if you have a set of additional rigging tools to simplify the manipulation of small objects and a well-thought-out strategy in the sequence of technological operations. It also greatly simplifies the situation in mass production, if it is possible to include various auxiliary details in the 3D model of the senor—for example, placement marks, keys, and guide cavities. It is unnecessary to mention that one can design and manufacture rigging tools on the same software and hardware as the MOX sensors described in the article. In addition, recent works [44,45,46,47] in the field of solid-state gas sensors witness that laser technique applied in our work may be used for creation/improving gas sensitive layers deposited on a YSZ/Pt microhotplate, which give additional impact to describing the article approach for rapid prototyping technology.

## 5. Conclusions

The main objective of this research was to demonstrate a rapid prototyping method for ceramic MEMS MOX gas sensors in a SMD SOT-23 package, and the reproducibility and stability of their properties. With this fabricated MOX gas sensors, we used the compromise between state-of-the-art technologies and materials: Perfect mechanical stability of YSZ ceramic membrane, fabricated by tape casting and sintering technology, high resolution of platinum metallization, printed by aerosol-jet system or sputtered by magnetron, and also qualitative geometrical parameters of bulk Al_2_O_3_ obtained by using laser micromilling. For the developed conception, MOX sensors mass production should be cost-effective and should not require any clean room technology. In this work we tested a way of fabrication of all gas sensor parts (including package), which can resist highly aggressive and harsh environmental conditions, like high humidity and temperature. The applied technologies have the potential to reduce the production costs of the final product and will simplify and speed up the whole production process chain. The described approach of developing and fabricating MOX sensors can lead to new impulses for applications in many different gas sensing tasks, such as medical, public security, and industrial safety. In particular, the developed technology will be interested in applications, which have small volume needs and are used in specific markets, which do not accept large financial investments for production infrastructure.
